# Chlorophyll Pigments of Olive Leaves and Green Tea Extracts Differentially Affect Their Antioxidant and Anticancer Properties

**DOI:** 10.3390/molecules28062779

**Published:** 2023-03-20

**Authors:** Abdülhadi Cihangir Uğuz, Javier Rocha-Pimienta, Sara Martillanes, María Garrido, Javier Espino, Jonathan Delgado-Adámez

**Affiliations:** 1 Department of Biophysics, School of Medicine, Karamanoğlu Mehmetbey University, Karaman TR-70200, Turkey; 2 Technological Agri-Food Institute (CICYTEX-INTAEX), Junta of Extremadura, Avda. Adolfo Suárez s/n, 06007 Badajoz, Spain; 3 Neuroimmunophysiology and Chrononutrition Research Group, Department of Physiology, Faculty of Science, University of Extremadura, 06006 Badajoz, Spain

**Keywords:** green tea, olive leaf, pro-oxidant, chlorophyll, bioactivity

## Abstract

Plant-based extracts possess biological potential due to their high content of phytochemicals. Nevertheless, photosynthetic pigments (e.g., chlorophylls) that are also present in plant extracts could produce undesirable pro-oxidant activity that might cause a negative impact on their eventual application. Herein, the phenolic content of olive leaf (OLE) and green tea (GTE) extracts was assayed, and their antioxidant and anticancer activities were evaluated before and after the removal of chlorophylls. Regarding phenolic content, OLE was rich in hydroxytyrosol, tyrosol as well as oleuropein, whereas the main compounds present in GTE were gallocatechin, epigallocatechin (EGC), epigallocatechin gallate (EGCG), gallocatechin gallate, and caffeine. Interestingly, fresh extracts’ antioxidant ability was dependent on phenolic compounds; however, the elimination of chlorophyll compounds did not modify the antioxidant activity of extracts. In addition, both OLE and GTE had high cytotoxicity against HL-60 leukemic cell line. Of note, the removal of chlorophyll pigments remarkably reduced the cytotoxic effect in both cases. Therefore, our findings emphasize the remarkable antioxidant and anticancer potential of OLE and GTE and suggest that chlorophylls are of paramount importance for the tumor-killing ability of such plant-derived extracts.

## 1. Introduction

In the last decades, plants have emerged as significant sources of phytochemical compounds, which have been used for applications in many fields, including the food industry and medicine. For instance, phytochemicals extracted from plants are employed for the design and development of multiple functional and/or nutraceutical ingredients. On the other hand, the use of medicinal plants for therapeutical or prophylactic purposes is based on their high content of antioxidants such as phenolic compounds [1]. According to epidemiological data, the consumption of fruits and vegetables greatly lowers the risk of suffering noncommunicable diseases, such as heart disease or cancer [2]. These potential benefits are attributed to their phytochemical components, which include but are not limited to vitamins, lycopene, carotenoids, and phenolic compounds.

Phenolics are secondary products involved in plant physiological functions. As phenolic compounds cannot be produced by the body, they must be incorporated into the diet along with other essential nutrients to benefit from their healthy attributes. This group of compounds possesses antioxidant activity, which allows them to prevent oxidative stress-mediated damage [1], evade tumor development [3], and display cardioprotective actions [4]. In parallel, phenolics have been also reported to exhibit pro-oxidant actions [5], which is worth further investigating to understand whether such pro-oxidant effects could influence their anticancer properties.

Phenolic compounds are abundantly present in different plant parts, including green tea (*Camellia sinensis* L.) [6] and olive leaves (*Olea europaea* L.) [7]. In this sense, olive leaves aqueous extract (OLE) is rich in hydroxytyrosol, tyrosol, oleuropein, quercetin, luteolin, caffeic and vanillic acids, ligstroside, and verbascoside [7]. Among them, oleuropein has been acknowledged as the active principle accountable for OLE’s antioxidant activity [8]. OLE also exhibits antiviral [9] and antimicrobial [10] properties. In addition, both hydroxytyrosol and oleuropein reportedly trigger apoptosis and hinder the proliferation of breast tumor cells [11]. As for green tea extract (GTE), catechins and caffeine are its major phenolic compounds [12]. Importantly, catechins possess anticancer capacity against numerous human tumor cell lines [13]. Particularly, epigallocatechin gallate (EGCG), epigallocatechin (EGC), and epicatechin gallate (ECG) may inhibit sustained proliferation and cell death resistance in lung cancer cells [14]. Additionally, EGCG combined with cancer preventive agents such as COX-2 inhibitors and retinoids synergistically enhanced the induction of apoptosis in diverse cancer cell lines [15]. Likewise, catechins may also find therapeutical applications because of their antimicrobial and anti-inflammatory effects [16]. 

On the other hand, photosynthetic pigments present in plant extracts may also display biological activity. In this sense, chlorophyll and its derivatives may be used as chemopreventive agents due to their antioxidant, antimutagenic, and pro-apoptotic activities [17,18]. At the same time, chlorophyll pigments may also exhibit pro-oxidant properties owing to their catalytic effect. In fact, the pro-oxidant activity of GTE on the photooxidation of marine oils was demonstrated to rely on its chlorophyll constituents [19]. However, catechins may also account for the pro-oxidant effect of GTE [5,20]. Thus, the putative anticancer ability of plant extracts may be linked to their pro-oxidant capacity. Herein, the potential antioxidant and anticancer activities of OLE and GTE were evaluated before and after the elimination of chlorophylls. Likewise, the contribution of individual phenolics to the biological activity of the plant extracts was also investigated.

## 2. Results and Discussion

### 2.1. Phenolic Content before and after Removal of Chlorophylls from Extracts

According to previous reports, phenolic compounds were extracted in the aqueous phase to optimize their preservation [21]. Potentially toxic solvents, e.g., methanol or hexane, were avoided [22] so that the extracts could be potentially applied in fields such as medicine or the agrifood industry. The purification of the extracts to obtain chlorophyll-free OLE and GTE produced a partial decrease in polyphenols content. In fact, the concentration of phenolic compounds was 2631.34 mg/L and 8205.94 mg/L in OLE and GTE, whereas it was 872.67 mg/L and 4680.52 mg/L in dechlorophyllized OLE and GTE. Thus, the yield of the purification process was 33.16% and 57.03% for OLE and GTE, respectively. 

Building upon these findings, HPLC was used to identify and quantify major individual phenolics of fresh OLE. As shown in Table 1, the main components were oleuropein (2173.52 mg/L) and hydroxytyrosol (445.73 mg/L), whereas tyrosol (12.09 mg/L) was found at a much lower concentration. These three bioactive substances represent more than 80% of phenolics present in OLE [23]. The elimination of chlorophylls from OLE significantly (*p* < 0.05) decreased the amount of oleuropein, hydroxytyrosol as well as tyrosol (Table 1).

**Table 1:** Major phenolics content of fresh and dechlorophyllized olive leaves (OLE) and green tea (GTE) extracts.

Compounds (mg/L)	Fresh OLE	Purified OLE
**Phenolic alcohol**		
Hydroxytyrosol	445.73 ± 3.10	187.30 ± 7.68 *
Tyrosol	12.09 ± 2.58	5.83 ± 0.16 *
**Secoiridoids derivatives**		
Oleuropein	2173.52 ± 110.80	679.53 ± 12.73 *
	**Fresh GTE**	**Purified GTE**
**Alkaloid**		
Caffeine	2232.52 ± 208.16	1483.30 ± 23.61 *
**Catechins**		
Gallocatechin	2536.82 ± 16.55	2215.18 ± 59.39 *
Epigallocatechin (EGC)	948.80 ± 36.29	802.00 ± 93.18
Epigallocatechin gallate (EGCG)	1829.27 ± 172.21	109.32 ± 3.27 *
Gallocatechin gallate	658.52 ± 18.44	70.73 ± 6.52 *

* p < 0.05 vs. its corresponding fresh extract (Student’s *t*-test). Data represent means ± SD of three sample replicates.

As for fresh GTE, the main bioactive molecules identified by HPLC were gallocatechin (2536.82 mg/L), caffeine (2232.52 mg/L), EGCG (1829.27 mg/L), EGC (948.80 mg/L), and gallocatechin gallate (658.52 mg/L) (Table 1), which fits into earlier findings that identified the very same compounds as the most abundant in GTE [23,24]. Again, the elimination of chlorophylls produced a noticeable reduction in the phenolic content, especially, in caffeine, EGCG, and gallocatechin gallate (*p* < 0.05; Table 1).

The rationale behind the drop in the concentration of polyphenols after purification could be attributed to their retention into the column, which impedes their full recovery, or to their transformation into simpler compounds. The latter has been previously described in green tea gallates, which are converted into gallic acid after column chromatography [25]. As for the retention of compounds, the latest passage of the eluent through the column to isolate the compounds of interest may not allow their full recovery. Consequently, the decline in the phenolic content because of the removal of photosynthetic pigments may also cause partial loss of the extracts’ bioactivity.

### 2.2. Antioxidant Properties of Extracts: Effect of Chlorophyll Pigments and Major Individual Phenolics

**Figure 1:**
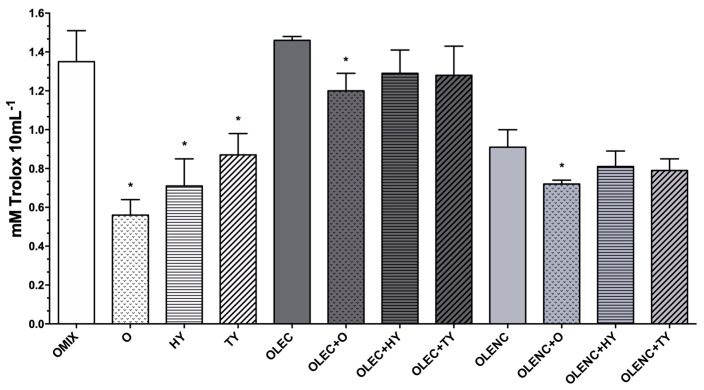
Antioxidant activity of olive leaves extract (OLE). Fresh (OLEC) and purified (OLENC) extracts, where indicated, were doped with pure oleuropein (O), hydroxytyrosol (HY), or tyrosol (TY). The concentrations are outlined in Table 2 of the Experimental Design. Results are shown as means ± SD of mM Trolox 10 mL^−1^. * *p* < 0.05 between doped extracts and their corresponding non-doped extract or between the individual compounds and OMIX (Tukey’s multiple test). OMIX: Mixture of OLE’s major phenolic compounds.

One of the most relevant benefits of green tea and olive leaf polyphenols is their antioxidant capacity, catechins, and oleuropein show the greatest antioxidant activity in such plant matrices [8,26]. Herein, fresh OLE presented high antioxidant activity (1.46 mmol Trolox 10 mL^−1^; Figure 1), which was 15-fold higher than that of other foodstuffs such as virgin olive oil [21]. The data of fresh extracts (OLEC) were normalized in order to be compared to the purified extracts (OLENC) and corroborate whether any potential reduction in antioxidant activity was caused by the loss of phenolics during the elimination of chlorophylls. Thus, after normalization, it was observed that the decline in the antioxidant capacity of the dechlorophyllized extract (OLENC; Figure 1) was not proportional (lower) to the loss of polyphenols because of the purification process, demonstrating that photosynthetic pigments did not affect OLE’s antioxidant potential.

Regarding standard compounds, they demonstrated a significant antioxidant action when administered alone, with oleuropein inducing the lowest activity and tyrosol the highest (Figure 1). The activity of the compounds rose when they were combined, but the combination (OMIX) did not exhibit synergistic actions since the sum of the separate activities was greater than the antioxidant effect of the mixed compounds (Figure 1). Furthermore, both fresh and purified OLE exhibited a similar trend when doped with any of the individual phenolics, though a statistically significant decrease was only found in oleuropein-doped extracts (OLEC + O and OLENC + O) (*p* < 0.05; Figure 1). These reductions supported earlier findings reporting dose-dependent pro-oxidant activity of polyphenols [5].

Given the antioxidative protection provided by OLE phenolics, they can be utilized for preventive and/or therapeutical applications. As a matter of fact, antioxidants isolated from olive leaves have been described to protect human red blood cells against oxidative stress-induced damage [27]. In addition, both oleuropein [28] and hydroxytyrosol [11] have been reported to display tumor-killing abilities in human breast cancer cells. Similar anticancer activity was also induced by polyphenolic antioxidants towards human leukemia cells, an effect dependent on their pro-oxidant properties [29].

**Figure 2:**
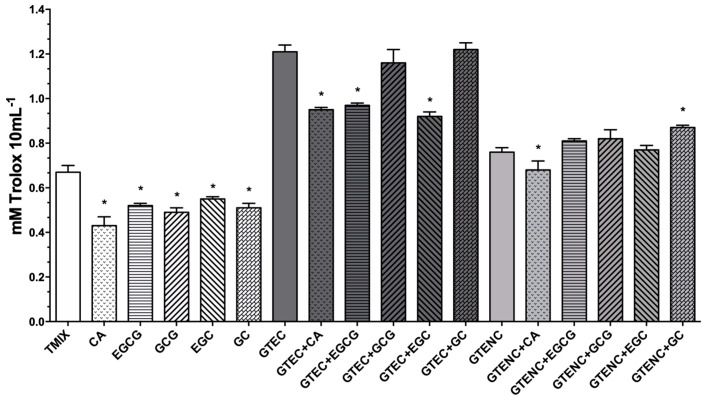
Antioxidant activity of green tea extract (GTE). Fresh (GTEC) and purified (GTENC) extracts, where indicated, were doped with pure caffeine (CA), epigallocatechin gallate (EGCG), gallocatechin gallate (GCG), epigallocatechin (EGC), or gallocatechin (GC). The concentrations are outlined in Table 3 of the Experimental Design. Results are shown as means ± SD of mM Trolox 10 mL^−1^. * *p* < 0.05 between doped extracts and their corresponding non-doped extract or between the individual compounds and TMIX (Tukey’s multiple test). TMIX: Mixture of GTE’s major phenolic compounds.

As for GTE, it also possesses elevated antioxidant capacity (1.2 mM Trolox 10 mL^−1^; Figure 2). In this case, it was detected that the reduction in the antioxidant capacity of the dechlorophyllized extract (GTENC; Figure 2) was similar to the loss of polyphenols owing to the purification process, demonstrating that photosynthetic pigments did not affect OLE’s antioxidant potential. The antioxidant activity shown by the purified GTE was therefore indicative of the action exclusively exerted by phenolics, ruling out the possibility that chlorophylls played a significant role in GTE’s antioxidant capacity.

On the other hand, every single pure GTE compound demonstrated antioxidant activity, with EGC producing the greatest antioxidant activity (*p* < 0.05; Figure 2). Although the mixture of GTE compounds (TMIX) had more antioxidant activity than the individual standards alone, this was insufficient to support an additive or synergistic relationship between them (Figure 2). Catechins have been proven to be more efficient than vitamins C, E, tocopherol, and carotenes in preventing DNA oxidative damage due to their powerful antioxidant properties [29,30]. Nevertheless, tea’s overall antioxidant capacity seems to be dependent on the combined action of several antioxidants, including phenolic acids and polyphenols, rather than one specific kind of polyphenol [30]. Regarding fresh GTE doped with pure compounds, its antioxidant activity was preserved or reduced in comparison with non-doped, fresh GTE (Figure 2). This effect was likely due to the pro-oxidant activity of phenolic compounds, which is dose-dependent [5]. In fact, rather than boosting the antioxidant action, a rise in the phenolic content of fresh GTE produced pro-oxidant activity, especially in the case of caffeine, EGCG, and EGC (*p* < 0.05, Figure 2). With the exception of the gallocatechin-doped extract (GTENC + GC), which markedly improved its antioxidant activity, the purified GTE doped with the standards exhibited the same tendency as the fresh GTE. The dechlorophyllized GTE (GTENC) doped with the standards exhibited the same tendency as the fresh GTE, except for the gallocatechin-doped extract (GTENC + GC), which markedly improved its antioxidant activity (*p* < 0.05, Figure 2). In a similar manner, saturation in the concentration of compounds following their addition to the purified GTE triggered pro-oxidant activity (i.e., caffeine; *p* < 0.05) or no modification in antioxidant capacity (i.e., EGCG) (Figure 2). Our findings suggested that the GTE could have a negative dose-dependent matrix effect because, in some situations, a rise in phenolic content has a detrimental impact on antioxidant capacity and, in other instances, can potentially have a pro-oxidant effect.

Potential therapeutic strategies against cancer may benefit from the role of GTE as an oxidative stress modulator. In fact, the pro-oxidant effect of green tea polyphenols may stimulate reactive oxygen species-mediated apoptosis of cancer cells, while their antioxidant actions may activate endogenous antioxidant defense systems in normal tissues that provide protection against tumor threat [20,31].

### 2.3. Anticancer Potential of Extracts: Impact of Chlorophylls and Major Individual Phenolics

**Figure 3:**
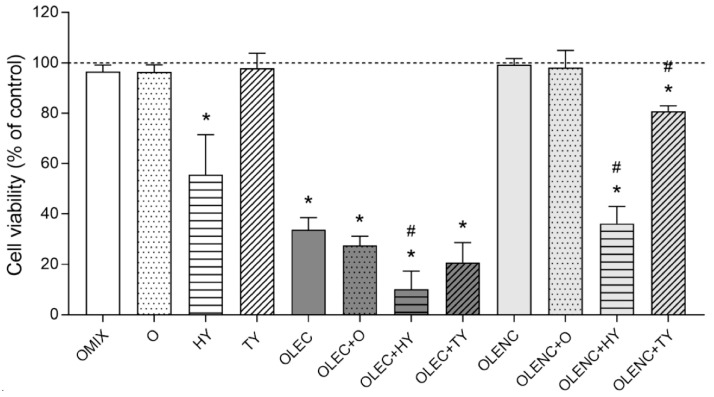
Antiproliferative activity of olive leaves extract (OLE). HL-60 cells were challenged with fresh (OLEC) and purified (OLENC) extracts in the absence or presence of pure oleuropein (O), hydroxytyrosol (HY), or tyrosol (TY) for 24 h at the concentrations indicated in Table 2 of the Experimental Design. Cells were also treated with OLE’s major individual phenolic compounds and the mixture of them (OMIX) at the same conditions. The dashed line represents control (untreated) samples. Results are shown as means ± SD of six independent experiments run in triplicate. * *p* < 0.05 vs. untreated samples (100% of cell viability; Tukey’s multiple test). ^#^ *p* < 0.05 between doped extracts and their corresponding non-doped extract (Tukey’s multiple test).

The anticancer or cytotoxic properties of plant extracts and their phytochemicals are well documented in the literature. The mechanisms of action of these molecules, the impact of other substances found in the extracts, and their interactions, however, need to be further explored and investigated. In this sense, we first demonstrated that fresh OLE (OLEC) possessed a remarkable cytotoxic effect against human promyelocytic leukemia HL-60 cells (~65% of cell death; *p* < 0.05; Figure 3). Interestingly, we found that the dechlorophyllized OLE (OLENC) caused a complete loss of its tumor-killing ability (Figure 3). This revealed that chlorophylls played a significant role in the anticancer action of OLE given that the purification procedure allowed for the recovery of around 33% of the phenolics detected in the extract. Chlorophylls have been previously mentioned as being important in chemoprevention because they may chelate certain substances that cause cancer, e.g., aflatoxin-B1 [17]. Moreover, a variety of mechanisms, including mutagen trapping, antioxidant, and antimutagenic actions, modification of xenobiotic metabolism, and activation of apoptosis have all been associated with the prevention of cancer by chlorophylls [18]. Despite the fact that chlorophylls’ direct actions on cancer cells have not yet been documented, it is evident that these substances may be very useful for developing novel cancer treatments.

Regarding the pure compounds contained in OLE, only hydroxytyrosol depicted cytotoxic activity against HL-60 cells (~50% of cell death; *p* < 0.05; Figure 3). OLE’s major phenolic compounds combination (OMIX) lacked cytotoxic action, most likely because the rest of the phenolics generated a negative matrix effect on hydroxytyrosol. Additionally, both fresh and purified extracts showed similar behaviors when doped with individual phenolic compounds. Thus, tyrosol slightly increased OLE’s cytotoxic capacity, while hydroxytyrosol largely enhanced such activity (~90% and ~65% of cell death in OLEC and OLENC, respectively; *p* < 0.05; Figure 3). Though its mechanisms of action are not entirely understood, hydroxytyrosol has well-known anticancer activity. In this regard, it has been proven that hydroxytyrosol possesses antiproliferative, pro-apoptotic, and anti-inflammatory properties [32], protects against UVB-induced oxidative DNA damage [33] and may help fight against diverse forms of cancer, including colon [34], prostate [35], breast [36], and blood [37] cancer.

**Figure 4:**
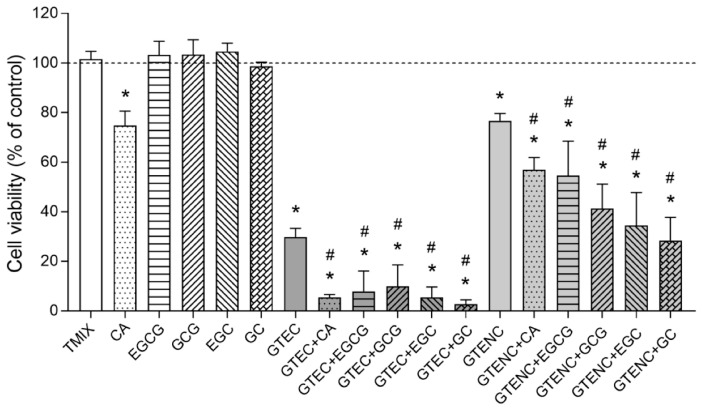
Antiproliferative activity of green tea extract (GTE). HL-60 cells were challenged with fresh (GTEC) and purified (GTENC) extracts in the absence or presence of pure caffeine (CA), epigallocatechin gallate (EGCG), gallocatechin gallate (GCG), epigallocatechin (EGC), or gallocatechin (GC). for 24 h at the concentrations indicated in Table 3 of the Experimental Design. Cells were also treated with GTE’s major individual phenolic compounds and the mixture of them (TMIX) at the same conditions. The dashed line represents control (untreated) samples. Results are shown as means ± SD of six independent experiments run in triplicate. * *p* < 0.05 vs. untreated samples (100% of cell viability; Tukey’s multiple test). ^#^ *p* < 0.05 between doped extracts and their corresponding non-doped extract (Tukey’s multiple test).

On the other hand, we showed that fresh GTE (GTEC) induced a noticeable cytotoxic action against HL-60 cells (~70% of cell death; *p* < 0.05; Figure 4). Remarkably, we observed that the removal of chlorophylls (GTENC) negatively affected the tumor-killing ability of the extract since its cytotoxic potential was reduced by more than half after the purification process (~30% of cell death; *p* < 0.05; Figure 4). These findings, which contrasted with GTE’s antioxidant ability, suggested that the extract’s anticancer potential is significantly influenced by chlorophylls. 

As for the individual compounds of GTE, caffeine was the only one exhibiting cytotoxic activity (~25% of cell death; *p* < 0.05; Figure 4). The mixture (TMIX) did not modify cell viability of HL-60 cells, likely because of a negative matrix effect of the other phenolics on caffeine. The anticancer potential of caffeine has been already posed owing to its ability to inhibit ATM and ATR kinases, which disrupts several DNA damage-responsive cell cycle checkpoints and bypasses tumor cells’ resistance to antitumor drugs [38]. Likewise, the direct interference of caffeine with DNA-PK activity and other DNA repair enzymes may also prevent the repair of DNA lesions in tumor cells [39].

Lastly, when both fresh (GTEC) and purified (GTENC) extracts were doped with any of the pure compounds, it was noticed that their tumor-killing potential was further improved (*p* < 0.05; Figure 4), which was seemingly due to a positive matrix effect. Gallocatechin was the compound with the greatest potentiating actions on the cytotoxic activity of fresh and purified GTE (*p* < 0.05; Figure 4). This result, together with the findings indicating that the individual compounds per se did not induce any cytotoxicity, supported the notion that catechins acted by amplifying the cytotoxic activity displayed by other molecules. As a matter of fact, the combination of green tea catechins such as EGCG with anticancer drugs synergistically induced apoptosis, gene expression changes, and anticancer effects [15], constrained tumor development in mice, and prevented tumor growth in xenograft animal models [40].

## 3. Materials and Methods

### 3.1. Plant Material

At a nearby business, olive leaves were collected (Badajoz, Spain). The samples were instantly brought to the lab in vented storage trays, vacuum-packaged (Gustav Müller vs. 100, Germany) in plastic bags (500 g), and frozen until their use (−80 °C). Care was taken during these procedures to prevent alterations to the material’s composition. A nearby grocery provided dried green tea leaves (Badajoz, Spain).

### 3.2. Preparation of Fresh and Dechlorophyllized Extracts

Fresh olive leaves were rinsed with distilled water and then partly dried for 12 min at 120 °C (model 210, Selecta^®^ P, Barcelona, Spain). Afterward, dried samples (green tea and olive leaves) were ground in a domestic knife mill to obtain particles (0.5–3.0 mm). Subsequently, bioactive compounds were extracted with water (1:10 *w/v*) at 118 °C for 15 min, and the samples were filtered and centrifuged to get rid of any solid residue. Finally, OLE and GTE were collected and stored at −80 °C until further analysis. 

A column (1.25 cm internal diameter and 20 cm height) packed with Toyopearl HW-40F was used to remove chlorophylls from extracts (Tosoh Bioscience LLC, Dorset, UK). Fresh extracts were added to the column after being dissolved in 80% (*v/v*) aqueous ethanol. Hexane was used to elute the column until all traces of the remaining green color were gone. After that, the column was washed with 80% (*v/v*) aqueous ethanol to retrieve the extracts without chlorophylls. Lastly, residual water was removed by lyophilization after the ethanol was evaporated under vacuum at 40 °C using a rotary evaporator.

### 3.3. Experimental Design

The extracts were examined both before and after being purified. To identify changes in the bioactive compounds contained in the extracts, the composition of phenolics as well as the antioxidant and anticancer properties were assessed. To evaluate the possible synergic or matrix effect of individual phenolic compounds present in both fresh and dechlorophyllized OLE and GTE, the extracts were diluted (1:10 *v/v*) and doped with standards of tyrosol (Sigma-Aldrich Chemie, Steinheim, Germany), hydroxytyrosol, and oleuropein (Extrasynthése, Genay, France), in the case of OLE, and with standards of EGCG (Adipogen, Liestal, Switzerland), EGC, gallocatechin gallate (Chengdu Biopurify Phytochemicals, Sichuan, China), gallocatechin (Extrasynthése), and caffeine (Enzo Biochem, Farmingdale, NY, USA), in the case of GTE. Table 2 and Table 3 provide a summary of the combinations that were examined.

### 3.4. Identification and Quantification of Phenolic Compounds by HPLC Analysis

Polyphenolic compound standards were prepared in methanol and kept at −20 °C in complete darkness. HPLC-grade methanol and acetonitrile (Fisher Chemical, Loughborough, UK) and P.A. grade formic acid (Panreac, Barcelona, Spain) were employed for the preparation of HPLC mobile phases. The HPLC analysis was performed in accordance with the procedures and parameters outlined by Cabrera-Bañegil et al. [41]. Using an Agilent 1100 HPLC system (Hewlett-Packard, Waldbronn, Germany) with a diode array detector (DAD) and fluorescence detector (FLD), the principal phenolic compounds were analyzed. A Gemini-NX C18 column (150 × 4.6 mm i.d., 3 µm thickness, Phenomenex) was also utilized.

**Table 2:** Combinations of individual phenolic compounds with both fresh and purified OLE.

	Oleuropein (mg/kg)	Tyrosol (mg/kg)	Hydroxytyrosol (mg/kg)	Abbreviations
**Multi-standard**	2000	2000	2000	OMIX
**Individual standards**	2000	-	-	O
	-	2000	-	TY
	-	-	2000	HY
**Fresh extract**	-	-	-	OLEC
	2000	-	-	OLEC + O
	-	2000	-	OLEC + TY
	-	-	2000	OLEC + HY
**Purified extract**	-	-	-	OLENC
	2000	-	-	OLENC + O
	-	2000	-	OLENC + TY
	-	-	2000	OLENC + HY

### 3.5. Measurement of OLE and GTE’s Antioxidant Activities

The ABTS^•+^ method was used to assess the extracts’ antioxidant capability [42]. In summary, several combinations of OLE and GTE samples (see Table 2 and Table 3) were combined with 100 μL of ABTS solution (2.2’-azinobis (3-ethylbenzoithiazolone 6-sulphonate), and these mixes were placed into 96-well microtiter plates. The absorbance was measured at 730 nm and the results were expressed as mmol Trolox 10 mL^−1^ using a calibration curve of Trolox.

### 3.6. Cell Culture and Determination of Anticancer Potential

Human promyelocytic leukemia HL-60 cells (ECACC No. 88120805; Dorset, UK) were cultured in RPMI 1640 medium (HyClone, Barcelona, Spain) supplemented with 2 mM L-glutamine, 10% heat-inactivated fetal bovine serum, 100 U/mL penicillin/streptomycin (Gibco, Barcelona, Spain). Cells were kept under a humidified atmosphere containing 5% CO_2_ at 37 °C.

Using the CellTiter 96^®^ AQueous One Solution Cell Proliferation Assay (Promega, Madrid, Spain), the cytotoxic effects of OLE and GTE were evaluated on HL-60 cells, as described elsewhere [43]. The cell viability was determined as percentage of control values (untreated samples).

**Table 3:** Combinations of individual phenolic compounds with both fresh and purified GTE.

	Caffeine (mg/kg)	Gallocatechin (mg/kg)	Epigallocatechin (mg/kg)	Epigallocatechin Gallate (mg/kg)	Gallocatechin Gallate (mg/kg)	Abbreviations
**Multi-standard**	2000	2000	2000	2000	2000	TMIX
**Individual standards**	2000	-	-	-	-	CA
	-	2000	-	-	-	GC
	-	-	2000	-	-	EGC
	-	-	-	2000	-	EGCG
	-	-	-	-	2000	GCG
**Fresh extract**	-	-	-	-	-	GTEC
	2000	-	-	-	-	GTEC + CA
	-	2000	-	-	-	GTEC + GC
	-	-	2000	-	-	GTEC + EGC
	-	-	-	2000	-	GTEC + EGCG
	-	-	-	-	2000	GTEC + GCG
**Purified extract**	-	-	-	-	-	GTENC
	2000	-	-	-	-	GTENC + CA
	-	2000	-	-	-	GTENC + GC
	-	-	2000	-	-	GTENC + EGC
	-	-	-	2000	-	GTENC + EGCG
	-	-	-	-	2000	GTENC + GCG

### 3.7. Statistical Analysis

SPSS 18.0 statistical analysis software (SPSS Inc., Chicago, IL, USA) was used to analyze the results, which were expressed as means and standard deviations. Three replicates were carried out for each treatment or analysis unless otherwise indicated. The data were compared using one-way analysis of variance (ANOVA), and all tests were deemed statistically significant at *p* < 0.05. When significant differences were found, means were compared using Tukey’s test and Student’s t-test, as indicated in the corresponding legend.

## 4. Conclusions

Our findings highlighted the remarkable antioxidant and anticancer potential of OLE and GTE and suggested that chlorophylls are of paramount importance for the tumor-killing ability of such plant-derived extracts. Therefore, it is reasonable to assume that both OLE and GTE might be used as promising tools for future human anticancer research and therapy. However, further studies are warranted to deeply investigate the mechanism of action of these molecules and the interaction effect between them.

## Data Availability

The data are contained within this article.

## References

[1] Balasundram N., Sundram K., Samman S. (2006). Phenolic compounds in plants and agri-industrial by-products: Antioxidant activity, occurrence, and potential uses. Food Chem..

[2] Van’t Veer P., Jansen M.C., Klerk M., Kok F.J. (2000). Fruits and vegetables in the prevention of cancer and cardiovascular disease. Public Health Nutr..

[3] Moon Y.J., Wang X., Morris M.E. (2006). Dietary flavonoids: Effects on xenobiotic and carcinogen metabolism. Toxicol. Vitr..

[4] Kris-Etherton P.M., Hecker K.D., Bonanome A., Coval S.M., Binkoski A.E., Hilpert K.F., Griel A.E., Etherton T.D. (2002). Bioactive compounds in foods: Their role in the prevention of cardiovascular disease and cancer. Am. J. Med..

[5] Dintcheva N.T., Arrigo R., Baiamonte M., Rizzarelli P., Curcuruto G. (2017). Concentration-dependent anti-/pro-oxidant activity of natural phenolic compounds in bio-polyesters. Polym. Degrad. Stab..

[6] Rusak G., Komes D., Likić S., Horžić D., Kovač M. (2008). Phenolic content and antioxidative capacity of green and white tea extracts depending on extraction conditions and the solvent used. Food Chem..

[7] Talhaoui N., Taamalli A., Gómez-Caravaca A.M., Fernández-Gutiérrez A., Segura-Carretero A. (2015). Phenolic compounds in olive leaves: Analytical determination, biotic and abiotic influence, and health benefits. Food Res. Int..

[8] Škerget M., Kotnik P., Hadolin M., Hraš A.R., Simonič M., Knez Ž. (2005). Phenols, proanthocyanidins, flavones and flavonols in some plant materials and their antioxidant activities. Food Chem..

[9] Micol V., Caturla N., Pérez-Fons L., Más V., Pérez L., Estepa A. (2005). The olive leaf extract exhibits antiviral activity against viral haemorrhagic septicaemia rhabdovirus (VHSV). Antivir. Res..

[10] Sudjana A.N., D’Orazio C., Ryan V., Rasool N., Ng J., Islam N., Riley T.V., Hammer K.A. (2009). Antimicrobial activity of commercial *Olea europaea* (olive) leaf extract. Int. J. Antimicrob. Agents.

[11] Han J., Talorete T.P.N., Yamada P., Isoda H. (2009). Anti-proliferative and apoptotic effects of oleuropein and hydroxytyrosol on human breast cancer MCF-7 cells. Cytotechnology.

[12] Nishitani E., Sagesaka Y.M. (2004). Simultaneous determination of catechins, caffeine and other phenolic compounds in tea using new HPLC method. J. Food Compos. Anal..

[13] Fujiki H., Sueoka E., Watanabe T., Suganuma M. (2015). Synergistic enhancement of anticancer effects on numerous human cancer cell lines treated with the combination of EGCG, other green tea catechins, and anticancer compounds. J. Cancer Res. Clin. Oncol..

[14] Suganuma M., Okabe S., Kai Y., Sueoka N., Sueoka E., Fujiki H. (1999). Synergistic effects of (-)-epigallocatechin gallate with (-)-epicatechin, sulindac, or tamoxifen on cancer-preventive activity in the human lung cancer cell line PC-9. Cancer Res..

[15] Suganuma M., Saha A., Fujiki H. (2011). New cancer treatment strategy using combination of green tea catechins and anticancer drugs. Cancer Sci..

[16] Sinija V.R., Mishra H.N. (2009). Green tea: Health benefits. J. Nutr. Environ. Med..

[17] Egner P.A., Muñoz A., Kensler T.W. (2003). Chemoprevention with chlorophyllin in individuals exposed to dietary aflatoxin. Mutat. Res.-Fundam. Mol. Mech. Mutagen..

[18] Ferruzzi M.G., Blakeslee J. (2007). Digestion, absorption, and cancer preventative activity of dietary chlorophyll derivatives. Nutr. Res..

[19] Wanasundara U.N., Shahidi F. (1998). Antioxidant and pro-oxidant activity of green tea extracts in marine oils. Food Chem..

[20] Azam S., Hadi N., Khan N.U., Hadi S.M. (2004). Prooxidant property of green tea polyphenols epicatechin and epigallocatechin-3-gallate: Implications for anticancer properties. Toxicol. Vitr..

[21] Delgado-Adámez J., Franco Baltasar M.N., Ayuso Yuste M.C., Martín-Vertedor D. (2014). Oxidative stability, phenolic compounds and antioxidant potential of a virgin olive oil enriched with natural bioactive compounds. J. Oleo Sci..

[22] Japón-Luján R., Luque De Castro M.D. (2008). Liquid-liquid extraction for the enrichment of edible oils with phenols from olive leaf extracts. J. Agric. Food Chem..

[23] Kang J.H., Chung S.T., Go J.H., Row K.H. (2006). Separation of epigallocatechin gallate from Korean green tea by RP-HPLC. J. Liq. Chromatogr. Relat. Technol..

[24] Perva-Uzunalić A., Škerget M., Knez Ž., Weinreich B., Otto F., Grüner S. (2006). Extraction of active ingredients from green tea (Camellia sinensis): Extraction efficiency of major catechins and caffeine. Food Chem..

[25] Balentine D.A., Wiseman S.A., Bouwens L.C.M. (1997). The chemistry of tea flavonoids. Crit. Rev. Food Sci. Nutr..

[26] Vinson J.A., Dabbagh Y.A., Serry M.M., Jang J. (1995). Plant flavonoids, especially tea flavonols, are powerful antioxidants using an in vitro oxidation model for heart disease. J. Agric. Food Chem..

[27] Paiva-Martins F., Gordon M.H. (2001). Isolation and characterization of the antioxidant component 3,4-dihydroxyphenylethyl 4-formyl-3-formylmethyl-4-hexenoate from olive (*Olea europaea*) leaves. J. Agric. Food Chem..

[28] Shamshoum H., Vlavcheski F., Tsiani E. (2017). Anticancer effects of oleuropein. Biofactors.

[29] Sergediene E., Jönsson K., Szymusiak H., Tyrakowska B., Rietjens I.M.C.M., Čenas N. (1999). Prooxidant toxicity of polyphenolic antioxidants to HL-60 cells: Description of quantitative structure-activity relationships. FEBS Lett..

[30] Lee K.W., Lee H.J., Lee C.Y. (2002). Antioxidant activity of black tea vs. green tea. J. Nutr..

[31] Forester S.C., Lambert J.D. (2011). The role of antioxidant versus pro-oxidant effects of green tea polyphenols in cancer prevention. Mol. Nutr. Food Res..

[32] Bernini R., Merendino N., Romani A., Velotti F. (2013). Naturally occurring hydroxytyrosol: Synthesis and anticancer potential. Curr. Med. Chem..

[33] Guo W., An Y., Jiang L., Geng C., Zhong L. (2010). The protective effects of hydroxytyrosol against UVB-induced DNA damage in HaCaT cells. Phyther. Res..

[34] Manna C., Galletti P., Cucciolla V., Moltedo O., Leone A., Zappia V. (1997). The protective effect of the olive oil polyphenol (3,4-dihydroxyphenyl)-ethanol counteracts reactive oxygen metabolite-induced cytotoxicity in Caco-2 cells. J. Nutr..

[35] Quiles J.L., Farquharson A.J., Simpson D.K., Grant I., Wahle K.W.J. (2002). Olive oil phenolics: Effects on DNA oxidation and redox enzyme mRNA in prostate cells. Br. J. Nutr..

[36] Warleta F., Quesada C.S., Campos M., Allouche Y., Beltrán G., Gaforio J.J. (2011). Hydroxytyrosol protects against oxidative DNA damage in human breast cells. Nutrients.

[37] Nousis L., Doulias P.T., Aligiannis N., Bazios D., Agalias A., Galaris D., Mitakou S. (2005). DNA protecting and genotoxic effects of olive oil related components in cells exposed to hydrogen peroxide. Free Radic. Res..

[38] DeFrank J.S., Tang W., Powell S.N. (1996). p53-null cells are more sensitive to ultraviolet light only in the presence of caffeine. Cancer Res..

[39] Block W.D., Merkle D., Meek K., Lees-Miller S.P. (2004). Selective inhibition of the DNA-dependent protein kinase (DNA-PK) by the radiosensitizing agent caffeine. Nucleic Acids Res..

[40] Fujiki H., Suganuma M. (2012). Green tea: An effective synergist with anticancer drugs for tertiary cancer prevention. Cancer Lett..

[41] Cabrera-Bañegil M., Pérez-Nevado F., Montaño A., Pleite R., Martín-Vertedor D. (2018). The effect of olive fruit maturation in Spanish style fermentation with a controlled temperature. LWT.

[42] Turoli D., Testolin G., Zanini R., Bellù R. (2004). Determination of oxidative status in breast and formula milk. Acta Paediatr..

[43] Gutiérrez-Tarriño S., Espino J., Luna-Giles F., Rodríguez A.B., Pariente J.A., Viñuelas-Zahínos E. (2021). Synthesis, characterization and antiproliferative evaluation of pt(Ii) and pd(ii) complexes with a thiazine-pyridine derivative ligand. Pharmaceuticals.

